# Influence of an inclined magnetic field and heat and mass transfer on the peristaltic flow of blood in an asymmetric channel

**DOI:** 10.1038/s41598-023-30378-5

**Published:** 2023-04-07

**Authors:** M. A. Abdelhafez, A. M. Abd-Alla, S. M. Abo-Dahab, Yasmine Elmhedy

**Affiliations:** 1 Department of Mathematics, Faculty of Science, Sohag University, Sohag, Egypt; 2 Department of Mathematics, Faculty of Science, South Valley University, Qena, Egypt

**Keywords:** Biophysics, Materials science, Mathematics and computing

## Abstract

This article presents a theoretical study on heat and mass transfer analysis of the peristaltic flow of blood conveying through an asymmetric channel in the presence of inclined to the magnetic field. The effects of ratio of relaxation to retardation times, non-uniform parameter, the non-dimensional amplitude, Hartman number and phase difference have been taken into account. The governing coupled non-linear partial differential equations representing the flow model are transmuted into linear ones by assuming that the wave is very long with a small Reynolds number. The converted mathematical formulations are solved analytically via the Mathematica software. Analytical expressions for the dimensionless velocity profiles of fluid, temperature, concentration, pressure gradient, increase in pressure, heat transfer coefficient and shear stress of the blood are derived. The velocity, temperature, concentration, pressure gradient, increase in pressure, heat transfer coefficient and shear stress were calculated numerically for different values of the parameters, which were represented graphically and find their physical meaning.

## Introduction

In the human vascular system, the heart is the building block organ which pumps oxygenated blood to the body and deoxygenated blood to the lungs through the blood vessels (arteries, veins, and capillaries). For a healthy life cycle, the active and energetic functioning of the heart is necessary. In modern days, one of the most common causes of death in the world is cardiovascular diseases, like arteriosclerosis and post-stenotic dilation. Atherosclerosis (medically called stenosis) in a blood vessel is the partial occlusion of the blood flow region in the vessel by the accumulation of atherosclerotic plaques due to the deposits of fat, cholesterol, calcium, and other harmful material. Over the time, stenosis solidifies and make arterial wall rigid, inflexible, and constricts the blood vessel which limits the oxygenated blood supply to the organs and other parts of the body, and leads to severe complications, including myocardial infarction, strokes, angina pectoris, and cerebral strokes. An aneurysm or dilatation refers to a debilitating of a blood vessel wall that generates a hump, or enlargement, of the vessel. In a two dimensional channel the vital principles of peristaltic pumping has been studied in Jaffrin and Shapiro^1^ and values of various parameters that governing the flow are clarified. Influence of long wavelength at low values of Reynolds number on the peristaltic flow has been illustrated in Manton^2^. In the closed form solutions, the impact of heat transfer in the presence of a magnetic field on the peristaltic transport is examined in Akram and Nadeem^3^. In an asymmetric channel with porous medium, the peristaltic transport of Phan-Thien-Tanner fluid is investigated by Vajravelu et al.^4^. Srinivas et al.^5^, have been studying the effect of mass and heat transfer on MHD peristaltic flow via porous medium. In an inclined asymmetric, Vajravelu el al.^6^ are discussed the peristaltic flow of a conducting Jeffrey fluid. In a tube with an endoscope, the influence of radially magnetic field on peristaltic transport of Jeffery fluid investigated by Abd-Alla el at.^7^. In two dimensional flow of Williamson fluid, the effect of Newtonian and Joule Heating are illustrated by Hayat et al.^8^. Srinivas et al.^9^ discussed the peristaltic flow of a Newtonian fluid under heat transfer and porous medium in a vertical channel. Rajvanshi and Wasu^10^ study the MHD squeezing flow under heat transfer by using Brinkman model. In asymmetric channel, the partial slip is investigated on the peristaltic flow of Williamson fluid by Akram et al.^11^. For a couple stress fluid, the simulated peristaltic transport of chyme in the small intestine is discussed in Akbar and Nadeem^12^. In a vertical annulus, the peristaltic transport of limousine fluid under mass and heat transfer is studied by Akbar and Nadeem^13^. In drug delivery systems, the applications of nanofluids peristaltic transport is illustrated in Tripathi and Be'g^14^. Ojjela et al.^15^, are investigated under the presence of a magnetic field between two layers of a porous medium, the influence of thermophoresis on an unsteady two-dimensional laminar incompressible mixed convective chemically reacting transport and heat transfer Jeffery fluid. Under a uniform, normal magnetic field, the heat transfer on the peristaltic magnetohydrodynamic flow of the Jeffery fluid is illustrated via a porous medium in a vertical echelon channel by Krishna et al.^16^. In asymmetric channel, the impact of temperature independent viscosity is addressed on the peristaltic transport of Jeffery fluid by Hasona et al.^17^. The nonlinear radiative peristaltic flow of Jeffery nanofluid in a vertical asymmetric channel is discussed in Hayat et al.^18^. The impact of aligned magnetic and properties of channel wall, are addressed on the peristaltic flow of a Jeffery nanofluid under heat and mass transfer by Sucharitha et al.^19^. Ramesh and Devakar^20^ have examined Effects of Heat and Mass Transfer on the Peristaltic Transport of MHD Couple Stress Fluid through Porous Medium in a Vertical Asymmetric Channel. It is explored in Javed et al.^21^ the influence of elastic wall on peristaltic transport in an asymmetric channel. Saleem et al.^22^ are investigating the effect of inclined magnetic and velocity second boundary conditions into the peristaltic flow of a Jeffery fluid under heat and mass transfer in asymmetric channel. Through a non-uniform channel, the peristaltic flow of non-Newtonian fluid is inspected in Imran et al^23^. In the presence of heat transfer, the MHD peristaltic flow of Jeffery fluid in the compliant walled channel is addressed by Javed et al.^24^. Through a porous media channel, the influence of chemical reaction and magnetohydrodynamic in the peristaltic transport of a non-Newtonian Jeffery fluid is inspected by Abbas et al.^25^.

Recently, Abo-Dahab et al.^26^ discussed the double-diffusive peristaltic MHD Sisko nanofluid flow through a porous medium in presence of non-linear thermal radiation, heat generation/absorption, and Joule heating^27^ investigated the heat and mass transfer in a peristaltic rotating frame Jeffrey fluid via porous medium with chemical reaction and wall properties.

Most of the studies mentioned above were focused mainly on analyzing the MHD flow of blood through the an asymmetric channel with the heat and mass transfer phenomenon. Therefore, the novelty of the current research article is to explore the impact of magnetic field on blood in an asymmetric channel with the heat and mass transfer effect. The governing equations have been modeled under the assumption that the wave is very long with a small Reynolds number. This problem has been solved analytically under certain boundary conditions and obtaining analytical solution of velocity, temperature, concentration, pressure gradient, increase in pressure, heat transfer coefficient and shear stress, which were calculated numerically at different values of physically important parameters. It was possible to represent these results graphically and find the physical meaning for them.

## Formulation of the problem

**Figure 1:**
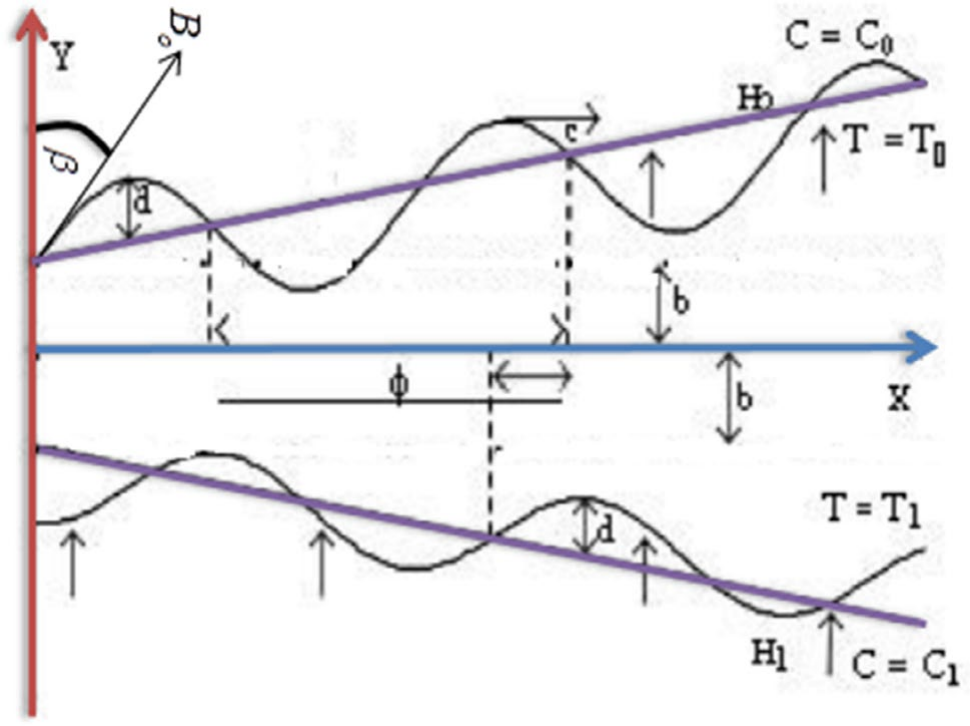
Schematic of the problem.

The model demonstrates the peristaltic transport of a viscous liquid through tapered horizontal channels of asymmetric dimensions of infinite length. The asymmetry in the flow is due to the propagation of peristaltic waves of different amplitudes and phases on the walls of the channel, assuming that the liquid is under the influence of a magnetic field inclined to the vertical is constant $B_{0}$ and that the flow is produced by trains propagating with steady speed c along the tapered asymmetric channel walls as shown in Fig. 1.

The geometry of the wall surface is defined as

1 $$ Y = \overline{{H_{2} }} = b + m^{\prime}\overline{X} + d\sin \left[ {\frac{2\pi }{\lambda }\left( {\overline{X} - ct} \right)} \right] $$

2 $$ Y = \overline{H}_{1} = - b - m^{\prime}\overline{X} - d\sin \left( {\frac{2\pi }{\lambda }(\overline{X} - c\overline{t} ) + \varphi } \right) $$

where b is the half-width of the channel, d is the wave amplitude, c is the phase speed of the wave and $\left(<1\right)$ is the non-uniform parameter, $\lambda$ is the wavelength, t is the time and X is the direction of wave propagation. The phase difference $\phi$ varies in the range $0\le \phi \le \pi , \phi =0$ corresponds to symmetric channel with waves out of phase and further b, d and $\phi$ satisfy the following conditions for the divergent channel at the inlet $d cos\left(\frac{\phi }{2}\right)\le b .$

It is assumed that the left wall of the channel is maintained at temperature ${T}_{0}$ while the right wall has temperature ${T}_{1}.$

The constitutive equations for an incompressible Jeffrey fluid are

3 $$ \overline{T} = - \overline{P} \overline{I} + \overline{S} $$

4 $$ \overline{S} = \frac{\mu }{{1 + \lambda_{1} \left( {\overline{\dot{r}} + \lambda_{2} \overline{\ddot{r}}} \right)}} $$

where $\overline{T }$ and $\overline{ S }$ are Cauchy stress tensor and extra stress tensor, respectively, p is the pressure,$\overline{I}$ is the identity tensor, $\lambda_{1}$ is the ratio of relaxation to retardation times, $\lambda_{2}$ is the retardation time $\ddot{r}$ is the shear rate and dots over the quantities indicate differentiation with respect to time.

In laboratory frame, the equations of continuity, momentum energy and concentration are described as follows

5 $$ \frac{{\partial \overline{U}}}{{\partial \overline{X}}} + \frac{{\partial \overline{V}}}{{\partial \overline{Y}}} = 0 $$

6 $$ \rho \left[ {\frac{\partial }{t} + \overline{U} \frac{\partial }{{\partial \overline{X} }} + \overline{V} \frac{\partial }{{\partial \overline{Y} }}} \right]\overline{U} = - \frac{{\partial \overline{P} }}{{\partial \overline{X} }} + \frac{{\partial \mathop {\overline{S} }\nolimits_{{\overline{X} \overline{X} }} }}{{\partial \overline{X} }} + \frac{{\partial \mathop {\overline{S} }\nolimits_{{\overline{X}\overline{Y}}} }}{{\partial \overline{Y} }} - \sigma \mathop {\mathop \beta \nolimits_{o} }\nolimits^{2} {\text{Cos}} \beta \left( {\overline{U} {\text{Cos}} \beta - \overline{V} {\text{Sin}} \beta } \right) $$

7 $$ \rho \left[ {\frac{\partial }{\partial t} + \overline{U} \frac{\partial }{{\partial \overline{X} }} + \overline{V} \frac{\partial }{{\partial \overline{Y} }}} \right]\overline{V} = - \frac{{\partial \overline{P} }}{{\partial \overline{Y} }} + \frac{{\partial \mathop {\overline{S} }\nolimits_{{\overline{X} \overline{Y} }} }}{{\partial \overline{X} }} + \frac{{\partial \mathop {\overline{S} }\nolimits_{{\overline{Y} \overline{Y} }} }}{{\partial \overline{Y} }} + \sigma \beta_{o}^{2} {\text{Sin}} \beta \left( {\overline{U} {\text{Cos}} \beta - \overline{V} {\text{Sin}} \beta } \right) $$

8 $$ \rho c_{p} \left[ {\overline{U} \frac{\partial }{{\partial \overline{X} }} + \overline{V} \frac{\partial }{{\partial \overline{Y} }}} \right]\overline{T} = K\left[ {\frac{{\partial^{2} }}{{\partial \overline{{X^{2} }} }} + \frac{{\partial^{2} }}{{\partial \overline{{Y^{2} }} }}} \right]\overline{T} + Q_{o} - \frac{\partial q}{{\partial y}} $$

9 $$ \left[ {\overline{U} \frac{{\partial \overline{C} }}{{\partial \overline{X} }} + \overline{V} \frac{{\partial \overline{C} }}{{\partial \overline{Y} }}} \right] = D_{m} \left[ {\frac{{\partial^{2} \overline{C} }}{{\partial \overline{{X^{2} }} }} + \frac{{\partial^{2} \overline{C} }}{{\partial \overline{{Y^{2} }} }}} \right] + \frac{{D_{m} K_{T} }}{{T_{m} }}\left[ {\frac{{\partial^{2} \overline{T} }}{{\partial \overline{{X^{2} }} }} + \frac{{\partial^{2} \overline{T} }}{{\partial \overline{{Y^{2} }} }}} \right] $$

where

10 $$ \begin{gathered} \overline{S}_{{\overline{X} \overline{X} }} = \frac{2\mu }{{1 + \lambda_{1} }}\left[ {1 + \lambda_{2} \left[ {\overline{|U} \frac{\partial }{{\partial \overline{X} }} + \overline{V} \frac{\partial }{{\partial \overline{Y} }}} \right]} \right]\frac{{\partial \overline{U} }}{{\partial \overline{X} }} \hfill \\ \overline{S}_{{\overline{X} \overline{Y} }} = \frac{\mu }{{1 + \lambda_{1} }}\left[ {1 + \lambda_{2} \left[ {\overline{U} \frac{\partial }{{\partial \overline{X} }} + \overline{V} \frac{\partial }{{\partial \overline{Y} }}} \right]} \right]\left[ {\frac{{\partial \overline{U} }}{{\partial \overline{Y} }} + \frac{{\partial \overline{V} }}{{\partial \overline{X} }}} \right] \hfill \\ \overline{S}_{{\overline{Y} \overline{Y} }} = \frac{2\mu }{{1 + \lambda_{1} }}\left[ {1 + \lambda_{2} \left[ {\overline{U} \frac{\partial }{{\partial \overline{X} }} + \overline{V} \frac{\partial }{{\partial \overline{Y} }}} \right]} \right]\frac{{\partial \overline{V} }}{{\partial \overline{Y} }} \hfill \\ \end{gathered} $$

where $\overline{U}$ and $\overline{V}$ are the velocity components in the laboratory frame ($\overline{X }$ ,$\overline{Y }$), k1 is the permeability of the porous medium, $\rho$ is the density of the fluid, p is the fluid pressure, *k* is the thermal conductivity,$\mu$ is the coefficient of the viscosity, *Q *$_{0}$ is the constant heat addition/absorption, *C *$_{p}$ is the specific heat at constant pressure, *σ* is the electrical conductivity, *g* is the acceleration due to gravity $\overline{T }$ is the temperature of the fluid, $\overline{C }$ is the concentration of the fluid, $T_{m}$ is the mean temperature, $D_{m}$ is the coefficient of mass diffusivity, and K $_{T}$ is the thermal diffusion ratio.

The relative boundary conditions are

11 $$ \begin{gathered} \begin{array}{*{20}c} {\overline{U} = o,} & {\begin{array}{*{20}c} {\overline{T} = T_{o} ,} & {\begin{array}{*{20}c} {\overline{C} = C_{o} } & {\begin{array}{*{20}c} {at} & {\overline{Y} = \overline{H}_{1} } \\ \end{array} } \\ \end{array} } \\ \end{array} } \\ \end{array} \hfill \\ \begin{array}{*{20}c} {\overline{U} = 0,} & {\overline{T} = T_{1} ,} & {\begin{array}{*{20}c} {\overline{C} = C_{1} } & {at} & {\overline{Y} = \overline{H}_{2} } \\ \end{array} } \\ \end{array} \hfill \\ \end{gathered} $$

Introducing a wave frame ($\overline{x }$,$\overline{y }$) moving with velocity c away from the fixed frame ($\overline{X} ,\,\overline{Y}$) by the transformation

12 $$ \overline{x} = \overline{X} - c\overline{t} ,\,\,\,\,\,\overline{y} = \overline{Y} ,\,\,\,\,\,\,\overline{u} = \overline{U} - c,\,\,\,\,\,\,\,\overline{v} = \overline{V} ,\,\,\,\overline{p} \left( x \right) = \overline{P} \left( {\overline{X} ,t} \right) $$

where $\stackrel{-}{u,}\overline{v }$ are the velocity components in the wave frame ($\overline{x }$ ,$\overline{y }$ ), $\overline{p }$ is pressures and $\overline{P }$ fixed frame of references. We introduce the following non-dimensional variables and parameters for the flow

13 $$ \begin{gathered} \begin{array}{*{20}c} {x = \frac{{\overline{x} }}{\lambda }} & {y = \frac{{\overline{y} }}{b}} & {\begin{array}{*{20}c} {\overline{t} = \frac{ct}{\lambda }} & {u = \frac{{\overline{u} }}{c}} & {v = \frac{{\overline{v} }}{\delta c}} & {\begin{array}{*{20}c} {S = \frac{{b\overline{S} }}{\mu c}} & {h_{1} = \frac{{\overline{{H_{1} }} }}{b}} & {P = \frac{{b^{2} \overline{P} }}{c\lambda \mu }} & {\delta = \frac{b}{\lambda }} \\ \end{array} } \\ \end{array} } \\ \end{array} \hfill \\ \begin{array}{*{20}c} {\theta = \frac{{\overline{T} - T_{o} }}{{T_{1} - T_{o} }}} & {\Theta = \frac{{\overline{C} - C_{o} }}{{C_{1} - C_{o} }}} & {{\text{Re}} = \frac{\rho cb}{\mu }} & {S_{c} = \frac{\mu }{{D_{m} \rho }}} \\ \end{array} \begin{array}{*{20}c} , & {S_{r} = \frac{{D_{m} \rho K_{T} \left( {T_{1} - T_{o} } \right)}}{{\mu T_{m} \left( {C_{1} - C_{o} } \right)}}} & , & {\varepsilon = \frac{d}{b}} \\ \end{array} \hfill \\ \begin{array}{*{20}c} {M = B_{o} b\sqrt {\frac{\sigma }{\mu }} } & {\Pr = \frac{{\mu C_{p} }}{K}} & {E_{c} = \frac{{c^{2} }}{{C_{p} \left( {T_{1} - T_{o} } \right)}}} & {\beta = \frac{{Q_{o} b^{2} }}{{\mu C_{p} \left( {T_{1} - T_{o} } \right)}}} \\ \end{array} \hfill \\ \end{gathered} $$

where $\varepsilon = \frac{d}{b}$ is the non-dimensional amplitude of channel,$\delta = \frac{b}{\lambda }$ is the wave number,$k_{1} = \frac{{\lambda m^{\prime}}}{b}$ is the non-uniform parameter, Re is the Reynolds number, M is the Hartmann number,$K = \frac{k}{{b^{2} }}$ Permeability parameter, Pr is the Prandtl number, $Ec$ is the Eckert number, β is the heat source/sink parameter, $Br \left( { = EcPr} \right)$ is the Brinkman number, $Sc$ Schmidt number and $S$ r Soret number.

## Solution of the problem

In view of the above transformations (12) and non-dimensional variables (13), Eqs. (5)–(9) are reduced to the following forms:

14 $$ \delta \left[ {\frac{\partial u}{{\partial x}} + \frac{\partial v}{{\partial y}}} \right] = 0 $$

15 $$ {\text{Re}} \delta \left[ {u\frac{\partial u}{{\partial x}} + v\frac{\partial u}{{\partial y}}} \right] = - \frac{\partial P}{{\partial x}} + \delta \frac{{\partial S_{xx} }}{\partial x} + \frac{{\partial S_{xy} }}{\partial y} - M^{2} {\text{Cos}}^{2} \beta \left( {u + 1} \right) $$

16 $$ {\text{Re}} \delta \left[ {u\frac{\partial v}{{\partial x}} + v\frac{\partial v}{{\partial y}}} \right] = - \frac{\partial P}{{\partial y}} + \delta^{2} \frac{{\partial S_{xy} }}{\partial x} + \delta \frac{{\partial S_{yy} }}{\partial y} + \delta M^{2} {\text{Sin}} \beta \left( {u{\text{Cos}} \beta - \delta v{\text{Sin}} \beta } \right) $$

17 $$ {\text{Re}} \delta \left[ {u\frac{\partial \theta }{{\partial x}} + v\frac{\partial \theta }{{\partial y}}} \right] = \frac{1}{\Pr }\left[ {\delta^{2} \frac{{\partial^{2} \theta }}{{\partial x^{2} }} + \frac{{\partial^{2} \theta }}{{\partial y^{2} }}} \right] + \beta + \frac{{N^{2} \theta }}{\Pr } $$

18 $$ {\text{Re}} \delta \left[ {u\frac{\partial \Theta }{{\partial x}} + v\frac{\partial \Theta }{{\partial y}}} \right] = \frac{1}{{S_{c} }}\left[ {\delta^{2} \frac{{\partial^{2} \Theta }}{{\partial x^{2} }} + \frac{{\partial^{2} \Theta }}{{\partial y^{2} }}} \right] + S_{r} \left[ {\delta^{2} \frac{{\partial^{2} \theta }}{{\partial x^{2} }} + \frac{{\partial^{2} \theta }}{{\partial y^{2} }}} \right] $$

where

19 $$ \begin{gathered} S_{xx} = \frac{2\delta }{{1 + \lambda_{1} }}\left[ {1 + \frac{{\lambda_{2} \delta c}}{d}\left[ {u\frac{\partial }{\partial x} + v\frac{\partial }{\partial y}} \right]} \right]\frac{\partial u}{{\partial x}} \hfill \\ S_{xy} = \frac{1}{{1 + \lambda_{1} }}\left[ {1 + \frac{{\lambda_{2} \delta c}}{d}\left[ {u\frac{\partial }{\partial x} + v\frac{\partial }{\partial y}} \right]} \right]\left[ {\frac{\partial u}{{\partial y}} + \delta^{2} \frac{\partial v}{{\partial x}}} \right] \hfill \\ S_{yy} = \frac{2\delta }{{1 + \lambda_{1} }}\left[ {1 + \frac{{\lambda_{2} \delta c}}{d}\left[ {u\frac{\partial }{\partial x} + v\frac{\partial }{\partial y}} \right]} \right]\frac{\partial v}{{\partial y}} \hfill \\ \end{gathered} $$

Applying long wave length approximation and neglecting the wave number along with low-Reynolds numbers.

Equations (14)–(18) become

20 $$ \frac{{\partial^{2} u}}{{\partial y^{2} }} = \left( {1 + \lambda_{1} } \right)\frac{\partial P}{{\partial x}} - M^{2} \left( {1 + \lambda_{1} } \right){\text{Cos}}^{2} \beta \left( {1 + u} \right) $$

21 $$ \frac{\partial P}{{\partial y}} = 0 $$

22 $$ \frac{{\partial^{2} \theta }}{{\partial y^{2} }} + N^{2} \theta = - \beta \Pr $$

23 $$ \frac{{\partial^{2} \Theta }}{{\partial y^{2} }} + S_{c} S_{r} \frac{{\partial^{2} \theta }}{{\partial y^{2} }} = 0 $$

The relative boundary conditions in dimensionless form are given by

24 $$ \begin{array}{*{20}c} {u = - 1,} & {\theta = 0,} & {\Theta = 0} & {\begin{array}{*{20}c} {at} & {y = h_{1} = - 1 - k_{1} x - \varepsilon {\text{Sin}} \left[ {2\pi x + \varphi } \right]} \\ \end{array} } \\ \end{array} $$

25 $$ \begin{array}{*{20}c} {u = - 1,} & {\theta = 1,} & {\Theta = 1} & {\begin{array}{*{20}c} {at} & {y = h_{2} = 1 + k_{1} x + \varepsilon {\text{Sin}} \left[ {2\pi x} \right]} \\ \end{array} } \\ \end{array} $$

The solutions of velocity, temperature and concentration with subject to boundary conditions (24) and (25) are given by

26 $$ u = \frac{ - 1}{B}\left[ {c + \left( {B - c} \right)\cos \left( {\frac{\sqrt B }{2}\left( {h_{1} + h_{2} - 2y} \right)} \right) \sec \left( {\frac{\sqrt B }{2}\left( {h_{1} - h_{2} } \right)} \right)} \right] $$

27 $$ \theta = \frac{1}{{N^{2} }}\left[ {2\left\{ {\left( {N^{2} + \beta Pr} \right) \cos \left( {\frac{N}{2}\left( {h_{1} - y} \right)} \right) - \beta \Pr \cos \left( {\frac{N}{2}\left( {h_{1} - 2 h_{2} + y} \right)} \right)} \right\} \csc \left( {N \left( {h_{1} - h_{2} } \right)} \right) \sin \left( {\frac{N}{2}\left( {h_{1} - y} \right)} \right)} \right] $$

28 $$\Theta =\frac{1}{2 \left({h}_{1}-{h}_{2}\right)}\left[2-\left[\frac{{-N}^{2}}{4 }\left({N}^{2}+\beta Pr\right) \mathrm{cos}\left(\frac{N}{2} \left({h}_{1}-y\right)\right)+\frac{{N}^{2}}{4} \beta \mathrm{Prcos}\left(\frac{N}{2}\left({h}_{1}-2 {h}_{2}+y\right)\right)\mathrm{csc}\left(N\left({h}_{1}-{h}_{2}\right)\right)\mathrm{sin}\left(\frac{N}{2}\left({h}_{1}-y\right)\right)+2\left[\frac{N}{2}\left({N}^{2}+\beta Pr\right)\mathrm{sin}\left(\frac{N}{2}\left({h}_{1}-y\right)\right)+\frac{N}{2}\beta \mathrm{Prsin}\left(\frac{N}{2}\left({h}_{1}-2{h}_{2}+y\right)\right)\right]\mathrm{csc}\left(N\left({h}_{1}-{h}_{2}\right)\right)\left(-N\right)\mathrm{cos}\left(\frac{N}{2}\left({h}_{1}-y\right)\right)+2\left[\left({N}^{2}+\beta Pr\right)\mathrm{cos}\left(\frac{N}{2}\left({h}_{1}-y\right)\right)-\beta \mathrm{Prcos}\left(\frac{N}{2}\left({h}_{1}-2{h}_{2}+y\right)\right)\right]\mathrm{csc}\left(N\left({h}_{1}-{h}_{2}\right)\right)\left(\frac{-{N}^{2}}{4}\right)\mathrm{sin}\left(\frac{N}{2}\left({h}_{1}-y\right)\right)\left(\frac{{s}_{c}{s}_{r}}{{N}^{2}}\right)\left({h}_{1}-{h}_{2}\right)\left({h}_{2}-y\right)\left({h}_{1}-y\right)\right]\right]$$

where

$$ B = - M^{2} \left( {1 + \lambda_{1} } \right) Cos^{2 } \beta $$

$$ c = B - \left( {1 + \lambda_{1} } \right)\frac{dP}{{dx}} $$

$$ N^{2} = - \frac{{b^{2} }}{{K\left( {\overline{T} - T_{0} } \right)}} $$

The coefficients of the heat transfer $Z{h}_{1}$ and $Z{h}_{2}$ at the walls $y={h}_{1} and y={h}_{2}$

29 $$ Zh_{1} = \theta_{y} h_{1x} , Zh_{2} = \theta_{y} h_{2x} $$

The instantaneous volum rate is defined as

30 $$ F = \int\limits_{{h_{1} }}^{{h_{2} }} {u\left( y \right)dy} $$

The gradient of the pressure is defined as

31 $$ \frac{dP}{{dx}} = \frac{{C_{1} \sqrt B }}{{\left( {1 + \lambda_{1} } \right)\left( {h_{2} - h_{1} } \right)}}\left[ {{\text{Sin}} \sqrt B h_{1} - {\text{Sin}} \sqrt B h_{2} } \right] + \frac{{C_{2} \sqrt B }}{{\left( {1 + \lambda_{1} } \right)\left( {h_{2} - h_{1} } \right)}}\left[ {{\text{Cos}} \sqrt B h_{2} - {\text{Cos}} \sqrt B h_{1} } \right] + \frac{B}{{\left( {1 + \lambda_{1} } \right)}} - \frac{BF}{{\left( {1 + \lambda_{1} } \right)\left( {h_{2} - h_{1} } \right)}} $$

The increase of the pressure is defined as

32 $$ \nabla p_{\lambda } = \int\limits_{0}^{2\pi } {\frac{dP}{{dx}}} dx $$

where

$$ C_{1} = \frac{{\left( { - B + c} \right)\left[ {{\text{Sin}} \sqrt B h_{2} - {\text{Sin}} \sqrt B h_{1} } \right]}}{{B\left[ {{\text{Sin}} \sqrt B \left( {h_{1} + h_{2} } \right)} \right]}} $$

$$ C_{2} = \frac{{\left( { - B + c} \right)\left( {{\text{Cos}} \sqrt B h_{2} - {\text{Cos}} \sqrt B h_{1} } \right)}}{{B\left( {{\text{Sin}} \sqrt B \left( {h_{1} + h_{2} } \right)} \right)}} $$

## Numerical results and discussion

In an asymmetric chaneel, we have obtained the closed form dimensionless expressions for the velocity $u$, temperature $\theta$, concentration $\Theta ,$ Heat Transfer Coefficient $Zh_{1}$ pressure gradient $\frac{dp}{{dx}}$, pressure rise $\Delta p_{\lambda }$ and tangential stress $s_{xy}$ are analyzed carefully. The physical variations in velocity profiles of both fluid, temperature, concentration, Heat Transfer Coefficient, pressure gradient, pressure rise $\Delta p_{\lambda }$ and tangential stress with respect to these sundry parameters are analyzed and discussed through the graphs 2–9.

**Figure 2:**
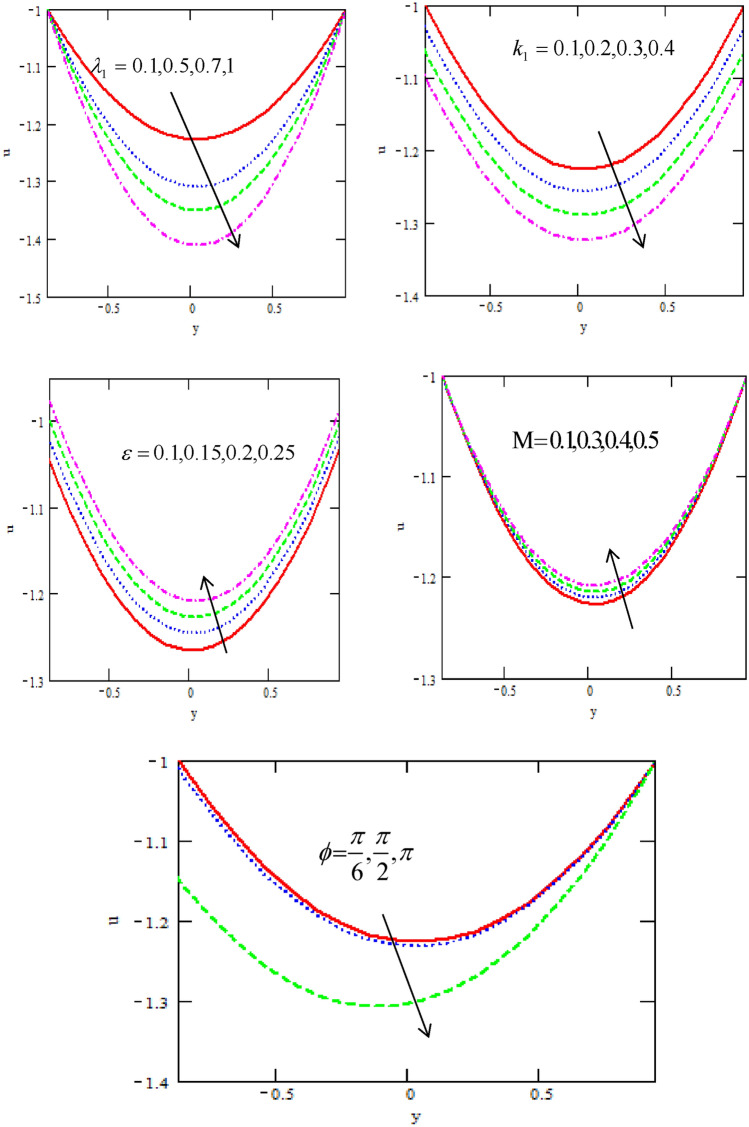
Variation of the velocity u concerning the axial-y with different values of $\lambda_{1} ,\,k_{1} ,\,\varepsilon ,\,{\rm M},\varphi$.

Figures 2 shows the variations of the velocity distribution $u$ with respect to the distance $y$ for different physical parameters of the ratio of relaxation to retardation times $\lambda_{1}$, non-uniform parameter $k_{1} ,$ the non-dimensional amplitude $\varepsilon ,$ Hartman number $M$ and phase difference $\varphi$. It is observed that the velocity distribution decreases with increasing of relaxation to retardation times, non-uniform parameter and phase difference while it increases with increasing of non-dimensional amplitude and Hartman number. It is noticed that the velocity satisfied the boundary conditions. On the otherhand, in the presence of a magnetic field, the influence of the assisting component of the magnetic force overcomes the impeding effect of the opposing component, resulting in a gradual increase in flow velocities.

**Figure 3:**
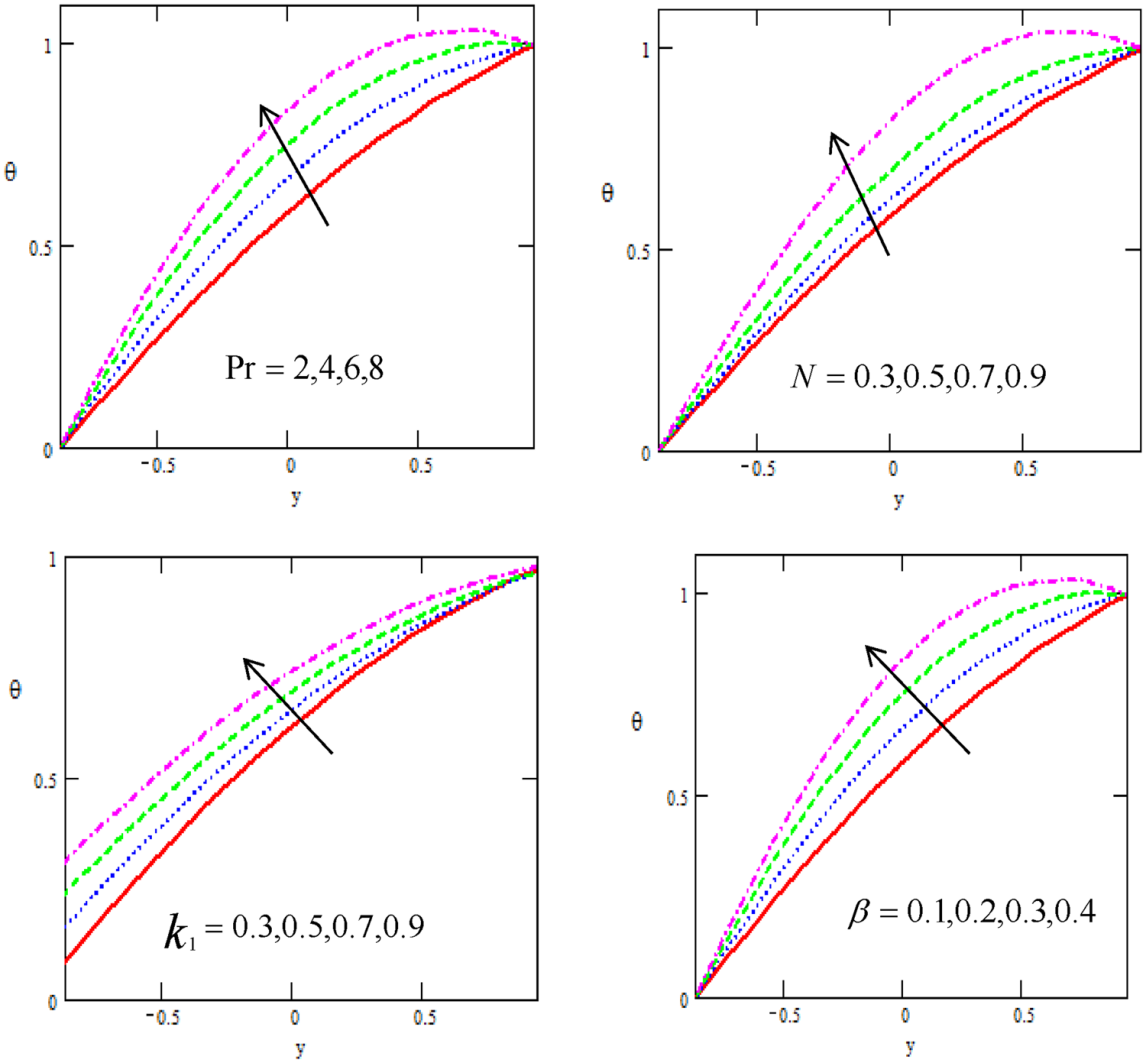
Variation of the Temperature $\theta$ concerning the axial-y with different values of $N,\,k_{1} ,\,\Pr ,\,\beta$.

Figures 3 shows the variations of the temperature distribution $\theta$ with respect to the distance $y$ for different physical parameters of the parameter number $N,$ Prandtl number $\Pr ,$ non-uniform parameter $k_{1}$ and heat source/sink $\beta .$ It is observed that the temperature distribution increases with increasing of $y - axis,$ as well it increases with increasing of parameter $N$, Prandtl number, non-uniform parameter and heat source/sink. It is noticed that the temperature satisfied the boundary conditions. These figures show that the fluid (blood) temperature increases for increasing values of the Prandtl number. This is because the higher values of the Prandtl number cause the fluid to have power thermal diffusivity and hence an increases in the fluid temperature.

**Figure 4:**
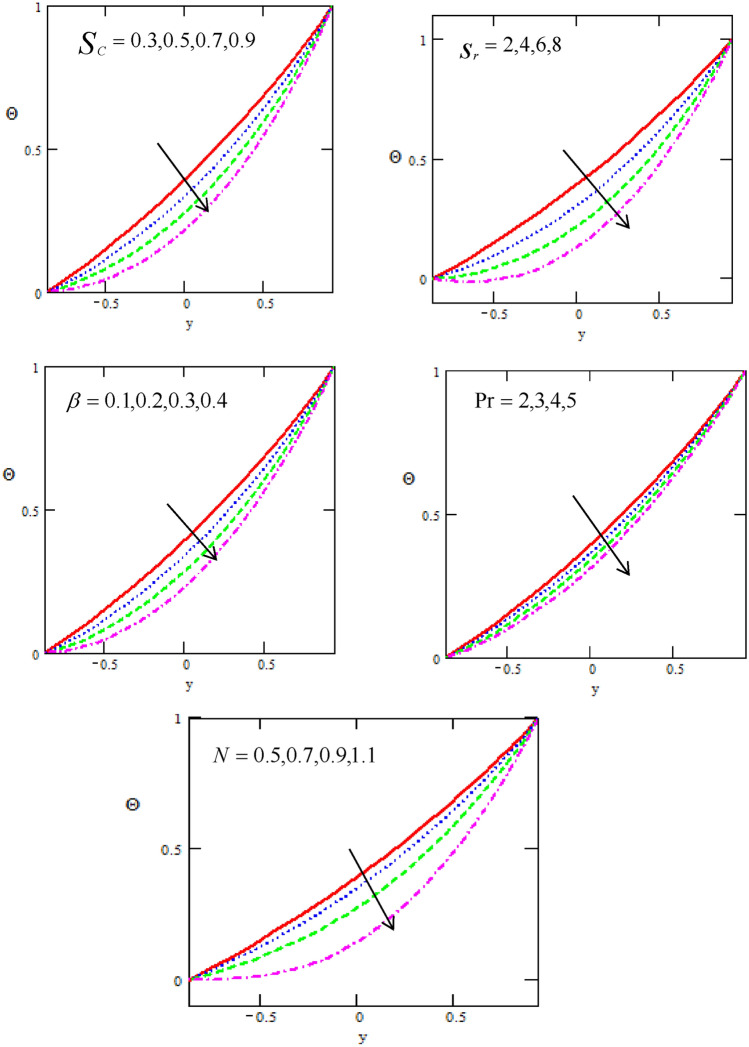
Variation of the Concentration $\Theta$ concerning the axial-y with different values of $Sc,Sr,\beta ,\Pr ,N$.

Figure 4 shows the variations of the Concentration $\Theta$ with respect to the distance $y$ for different physical parameters of the Schmidt number $Sc,$ Sort number $Sr,$ heat source/sink $\beta ,$ Prandtl number $\Pr$ and parameter number $N.$ It is observed that the Concentration distribution increases with increasing of $y - axis,$ as well it decreases with increasing of Schmidt number, Sort number, heat source/sink, Prandtl number and parameter number N, Prandtl number $\Pr$. It is noticed that the Concentration satisfied the boundary conditions.

**Figure 5:**
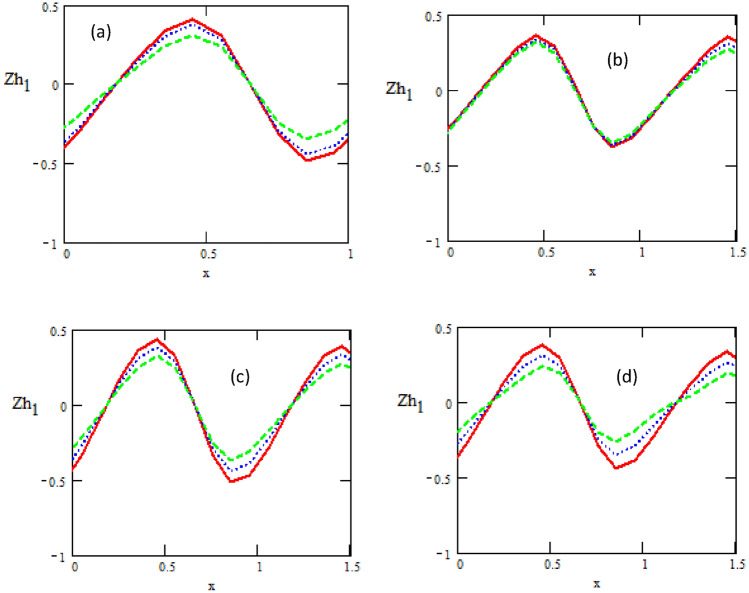
Variations of the heat transfer coefficient concerning the axial-x with different values of (**a**)$N = 0.1\,\_,\,0.3\,.\,..,\,0.5\, - -$, (**b**) $K_{1} = 0.01\,\_,\,0.05\,.\,..,\,0.09\, - -$, (**c**)$\beta = 0.01\,\_,\,0.05\,.\,..,\,0.09\, - -$, (**d**)$\Pr = 1\,\_,\,2\,.\,..,\,3 - -$.

**Figure 6:**
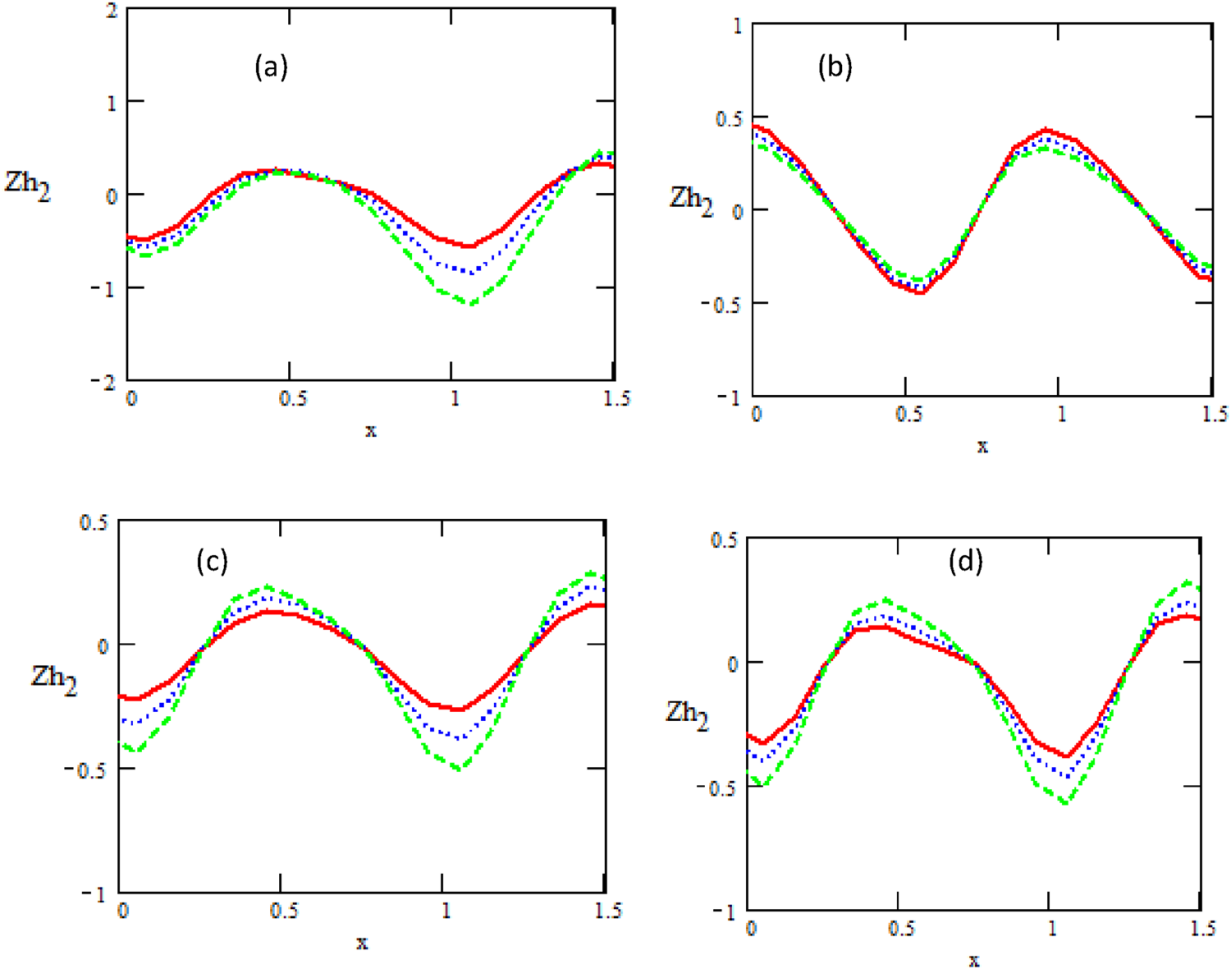
Variations of the heat transfer coefficient concerning the axial-x with different values of (**a**)$K_{1} = 0.1\,\_,\,0.3\,.\,..,\,0.5\, - -$, (**b**) $\Pr = 0.1\,\_,\,0.2\,.\,..,\,0.3 - -$, (**c**) $\varepsilon = 0.1\,\_,\,0.14\,.\,..,\,0.18\, - -$, (**d**)$N = 0.3\,\_,\,0.4\,.\,..,\,0.5 - -$.

Figures 5 and 6 show the variations of the heat transfer coefficients of the upper chanel $Zh_{1}$ and the heat transfer coefficients of the lower chanel $Zh_{2}$ with respect to the distance $x$ for different physical parameters of the parameter $N,$ non-uniform parameter $k_{1} ,$ heat source/sink $\beta ,$ and Prandtl number $\Pr .$ It is observed that the heat transfer coefficients of upper and lower chanel increase and decrease with increasing of parameter $N,$ non-uniform parameter, heat source/sink $\beta$ and Prandtl number $\Pr .$ It is noticed that the heat transfer coefficients are in oscillatory behavior, which may be due to peristalsis.

**Figure 7:**
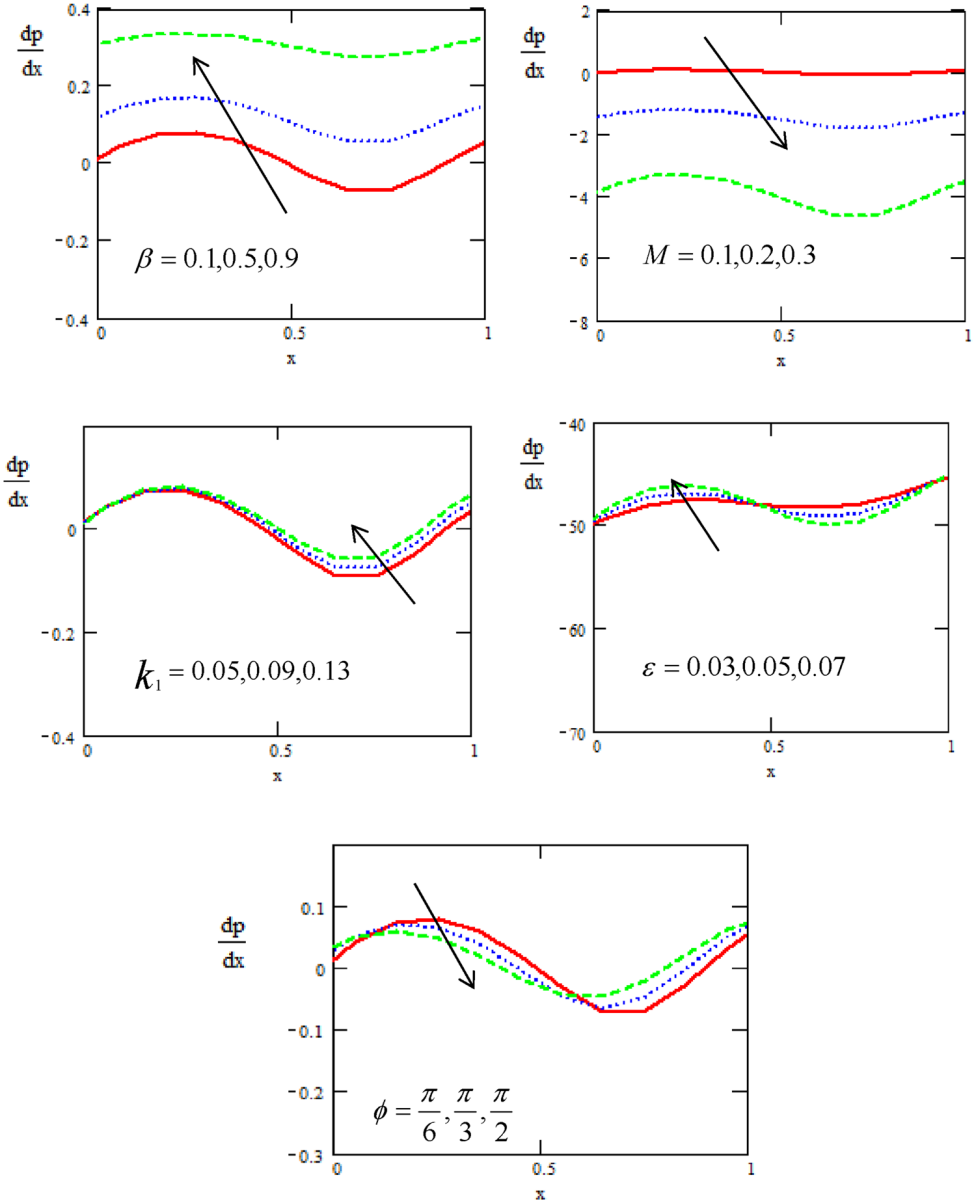
The gradient of Pressure concerning the axial-x with different values of $\varepsilon ,\beta ,M,\mathop k\nolimits_{1} ,\varphi$.

Figures 7 show the variations of the pressure gradient $\frac{dp}{{dx}}$ with respect to the distance $x$ for different physical parameters of the heat source/sink $\beta ,$ Hartman number $M,$ non-uniform parameter $k_{1} ,$ the non-dimensional amplitude $\varepsilon ,$ and phase difference $\varphi$. It is observed that the pressure gradient increases with increasing of, heat source/sink, while it is in oscillatory behavior in the whole range, which may be due to peristalsis.

**Figure 8:**
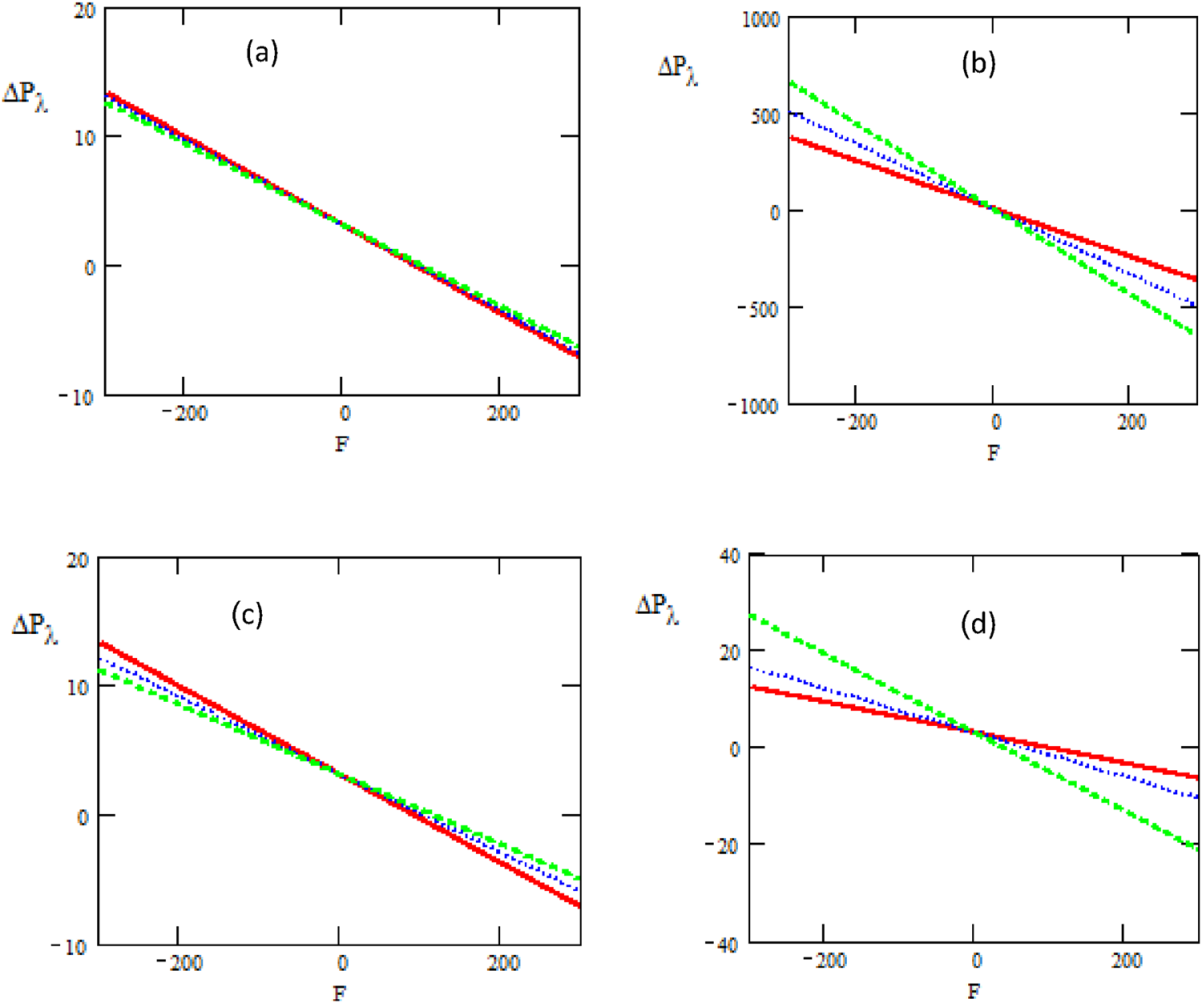
The pressure rise concerning the axial-F with different values of (**a**) $\beta = 0.1\,\_,\,0.2.\,..,\,0.3\, - -$, (**b**) $M = 0.6\_,\,0.7\,.\,..,\,0.8 - -$, (**c**) $K_{1} = 0.1\,\_,\,0.3\,.\,..,\,0.5\, - -$, (**d**)$\varepsilon = 0.1\,\_,\,0.5\,.\,..,\,0.9 - -$.

The influences of the phase difference $\varphi ,$ thermal slip $\gamma$ and viscosity parameter $\varepsilon$ are illustrated in Fig. 8. It is observed that the pressure rise $\Delta p_{\lambda }$ increases rapidly with the increase of heat source/sink $\beta ,$ Hartman number $M,$ non-uniform parameter $k_{1} ,$ the non-dimensional amplitude $\varepsilon .$ when $F \in ( - 300,\,0),$ while it decreases when $F \in (0,\,300).$ As expected, that pressure rise gives larger values for small volume flow rate $F$ and it gives smaller values for large volume flow rate. Moreover, the peristaltic pumping occurs in the region $- 300 \le F \le 300,$ otherwise augmented pumping occurs.

**Figure 9:**
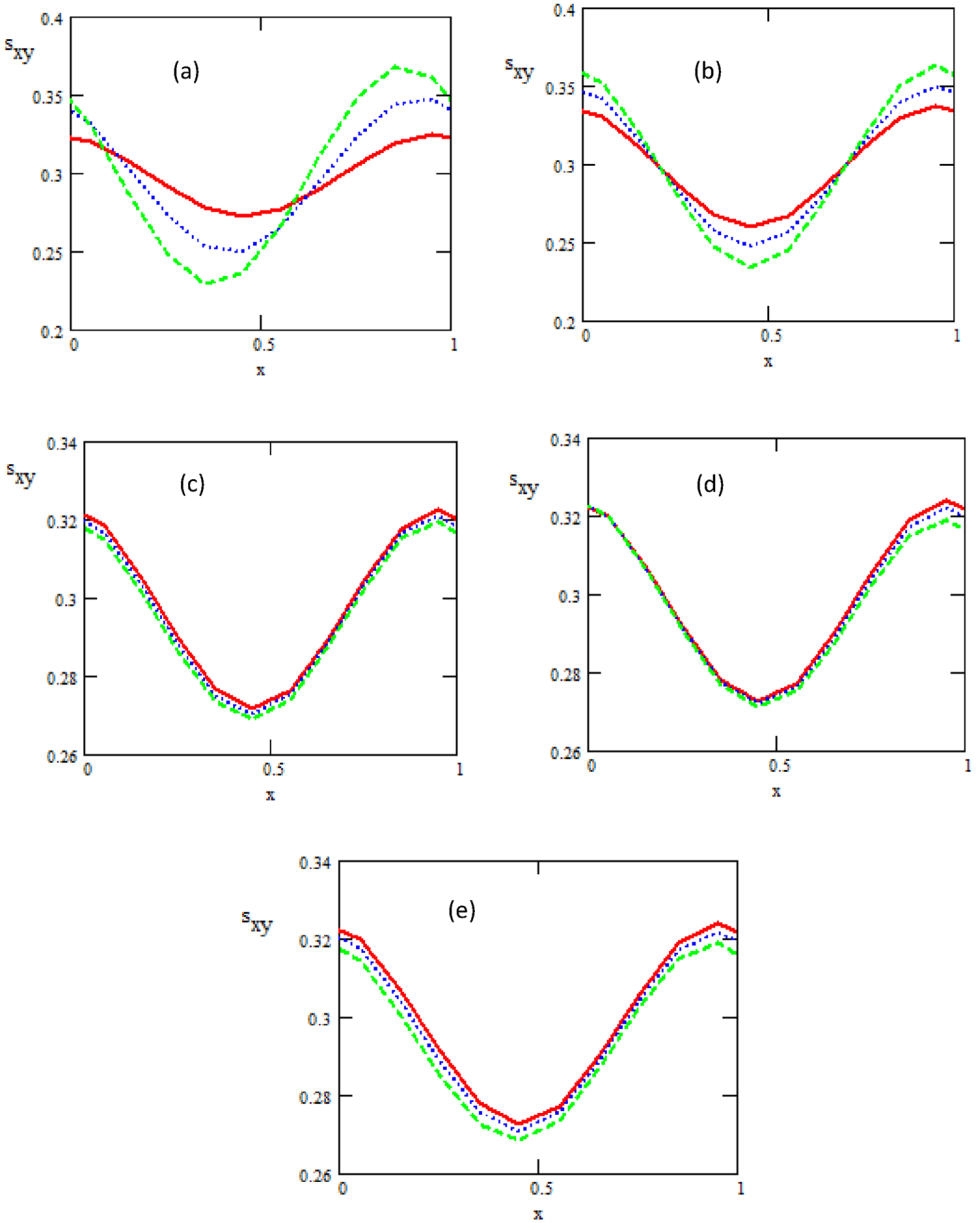
The Shear Stress concerning the axial-F with different values of (**a**) $\varphi = \frac{\pi }{6}\_\_,\,\frac{\pi }{3}\,.\,..,\,\frac{\pi }{2}\, - -$, (**b**) $\varepsilon = 0.3 - \,\_,\,0.4\,.\,..,\,0.5 - -$, (**c**) $\lambda_{1} = 1\,\_,\,2\,.\,..,\,3\, - -$, (**d**)$K_{1} = 0.1\,\_,\,0.6\,.\,..,\,1.1 - -$, (**e**)$M = 0.1\_,0.15....,0.2 - -$.

Figure 9 shows the variations of the tangential stress $s_{xy}$ with respect to $x -$ axis for various values of non-uniform parameter $k_{1} ,$ heat source/sink $\beta ,$ ratio of relaxation to retardation times $\lambda_{1,}$ phase difference $\varphi$ and Hartman number $M.$ From these figures, we observe that with the increase of non-uniform parameter and heat source/sink a tangential stress $s_{xy}$ is increasing, while it decreases with increasing of ratio of relaxation to retardation times, phase difference and Hartman number. It is noticed that one can observe the tangential stress is in oscillatory behavior, which may be due to peristalsis.

## Conclusion

The analytical solution has been obtained for velocity, temperature, concentration, pressure gradient, pressure rise, tangential stress and heat transfer coefficients have been discussed graphically. The major findings of the performed analysis are listed as follows:

1. An increase in $M$ while keeping all the other parameters fixed results in decrease of velocity.
2. It is observed that the concentration field increases with the increases in $N,\,\beta ,\,\Pr$ and $Sr.$
3. Heat transfer coefficients increase with increasing of $N,\,\Pr$ and $\beta$ in chanal.
4. The shear stress at the channel center and flow impedance are significantly reduced by increasing the magnetic field
5. Therefore, the judicious magnetic field can significantly regulate the motion of blood in the an asymmetric channel.
6. The obtained results of the present study may be useful in medical applications because they serve as useful estimations that can control the streaming blood as well as magnetic field.

## Data Availability

The datasets used and/or analyzed during the current study available from the corresponding author on reasonable request.
